# Recent advances in understanding of meiosis initiation and the apomictic pathway in plants

**DOI:** 10.3389/fpls.2014.00497

**Published:** 2014-09-23

**Authors:** Chung-Ju R. Wang, Ching-Chih Tseng

**Affiliations:** ^1^Institute of Plant and Microbial Biology, Academia Sinica, TaipeiTaiwan; ^2^Institute of Plant Biology, National Taiwan University, TaipeiTaiwan

**Keywords:** apomixis, meiosis initiation, RNA-directed DNA methylation, plant reproduction, ameiotic1

## Abstract

Meiosis, a specialized cell division to produce haploid cells, marks the transition from a sporophytic to a gametophytic generation in the life cycle of plants. In angiosperms, meiosis takes place in sporogenous cells that develop *de novo* from somatic cells in anthers or ovules. A successful transition from the mitotic cycle to the meiotic program in sporogenous cells is crucial for sexual reproduction. By contrast, when meiosis is bypassed or a mitosis-like division occurs to produce unreduced cells, followed by the development of an embryo sac, clonal seeds can be produced by apomixis, an asexual reproduction pathway found in 400 species of flowering plants. An understanding of the regulation of entry into meiosis and molecular mechanisms of apomictic pathway will provide vital insight into reproduction for plant breeding. Recent findings suggest that AM1/SWI1 may be the key gene for entry into meiosis, and increasing evidence has shown that the apomictic pathway is epigenetically controlled. However, the mechanism for the initiation of meiosis during sexual reproduction or for its omission in the apomictic pathway still remains largely unknown. Here we review the current understanding of meiosis initiation and the apomictic pathway and raised several questions that are awaiting further investigation.

## INTRODUCTION

Meiosis is an extremely important step in sexual reproduction. It is widely accepted that it evolved from mitosis and shares certain features with mitosis (Maynard Smith, 1978). Yet at least three meiosis-specific events make meiosis a specialized cell division: meiotic recombination and pairing between homologous chromosomes during prophase I, the suppression of sister-chromatid separation during the first meiotic division, and the absence of chromosome replication at the start of the second division (Kleckner, 1996). While these meiosis-specific events have been studied extensively, the mechanisms that switch mitosis into meiosis are still puzzling. In multicellular organisms, meiosis initiation takes place within multicellular organs; consequently, mechanisms that initiate meiosis must integrate developmental cues. In plants, the decision to start meiosis may also be connected with reproductive cell fate specification since plants do not have pre-determined germ lines. Thus, the switch of somatic fate to germinal cell fate and the mitosis–meiosis cell cycle transition occur sequentially during the development of reproductive organs (i.e., anthers and ovules; Ma, 2005). Importantly, these sexual processes can be replaced by the asexual apomictic pathway in which meiosis is bypassed or a mitosis-like division occurs to produce unreduced daughter cells, followed by the development of an embryo without fertilization, apomictic plants can then produce diploid seeds with identical genetic content to their maternal genome. This phenomenon is called apomixis that occurs naturally in some flowering plants (Barcaccia and Albertini, 2013). If apomixis is engineered into crops to produce clonal seeds, its application on agriculture will be broad and profound. Here, we review the current understanding of the cell cycle transition that directs sporogenous cells to leave the mitotic cell cycle and enter the meiotic program in higher plants and additionally discuss advances in the apomictic pathway.

## WHAT HAVE WE LEARNED FROM OTHER MODEL SPECIES ABOUT INITIATION OF MEIOSIS?

The cellular events during meiosis are evolutionarily conserved among species; however, the mechanisms controlling the initiation of meiosis are diverse (Pawlowski et al., 2007). The molecular controls elucidated to date involve signaling pathways, transcriptional and translational regulations of meiotic genes, and cyclin-dependent kinase (CDK) circuits. Although different mechanisms are adopted, the final readout is likely the activation of a specific cyclin–CDK complex to initiate the meiotic S phase. It was also suggested from many studies that the decision to start meiosis is made before the onset of the pre-meiotic S phase (Watanabe et al., 2001). Here, we first summarize the discoveries from several model species, and then discuss recent advances in plants.

The meiosis decision in single-celled yeasts is often cued by environmental conditions. In the budding yeast *Saccharomyces cerevisiae*, starvation induces expression of the *Initiator of Meiosis I* (*IME1*) gene, which encodes a transcription factor responsible for activating early meiotic genes (Chu et al., 1998). One of these target genes, *IME2*, which encodes a Ser/Thr protein kinase, promotes meiotic DNA replication by directly phosphorylating Rfa2, a subunit of replication protein A (Foiani et al., 1996; Clifford et al., 2005). Sic1, an inhibitor of the CDK (CDC28), is also phosphorylated by Ime2p and then leads to its degradation. Subsequently, CDC28, in conjunction with the B-type S phase cyclins, Clb5, and Clb6, triggers the initiation of the premeiotic S phase (Dirick et al., 1998; Stuart and Wittenberg, 1998).

In fission yeast *S. pombe*, a key transcription factor *STE11*, which is produced in response to environmental conditions, is responsible for early meiotic genes expression (Sugimoto et al., 1991). MEI2, an RRM-type RNA-binding protein, plays a crucial role in promoting entry into meiosis by regulating meiosis-specific mRNAs accumulation. During mitosis, MEI2 is inactivated by PAT1 kinase. Under meiosis-inducing conditions, this repression of MEI2 is released, allowing binding to and stabilization of meiosis-specific mRNAs at the G1 phase (Kitamura et al., 2001). In addition, this process reinforces stabilization also by sequestrating MMI1 protein, which function is to eliminate these meiotic mRNAs (Harigaya et al., 2006). Finally, CDC2 kinase binding with cyclin CIG2 is essential for entry into the pre-meiotic S phase (Borgne et al., 2002). Recently, protein S-palmitoylation, a lipid modification was also found to regulate the entry into meiosis (Zhang et al., 2013).

In mammals, meiosis is initiated at different stages of development in females and males (Bowles and Koopman, 2007). Mouse studies have revealed that retinoic acid (RA) produced during embryonic development can induce meiosis in both sexes. The level of RA is negatively regulated by the *Cyp26b1* enzyme that has RA degradation activity (Bowles et al., 2006; Koubova et al., 2006). *Stimulated by RA 8* (*Stra8*), a vertebrate-specific gene, is then induced by RA and is required for the transition to meiosis (Anderson et al., 2008). *Stra8* plays no role in the mitotic phases of embryonic germ-cell development, but in females it is required for pre-meiotic DNA replication and the subsequent events of meiotic prophase. On the other hand, *Dmrt1* represses *Stra8* transcription in the mitotic phase, thereby preventing meiosis (Matson et al., 2010).

From these studies, the mechanisms that initiate meiosis are very different, and more importantly, the genes involved share no similarity. No doubt different strategies evolved because of the different reproductive requirements of diverse organisms.

## THE DECISION OF MITOSIS–MEIOSIS SWITCH IN PLANTS

**FIGURE 1:**
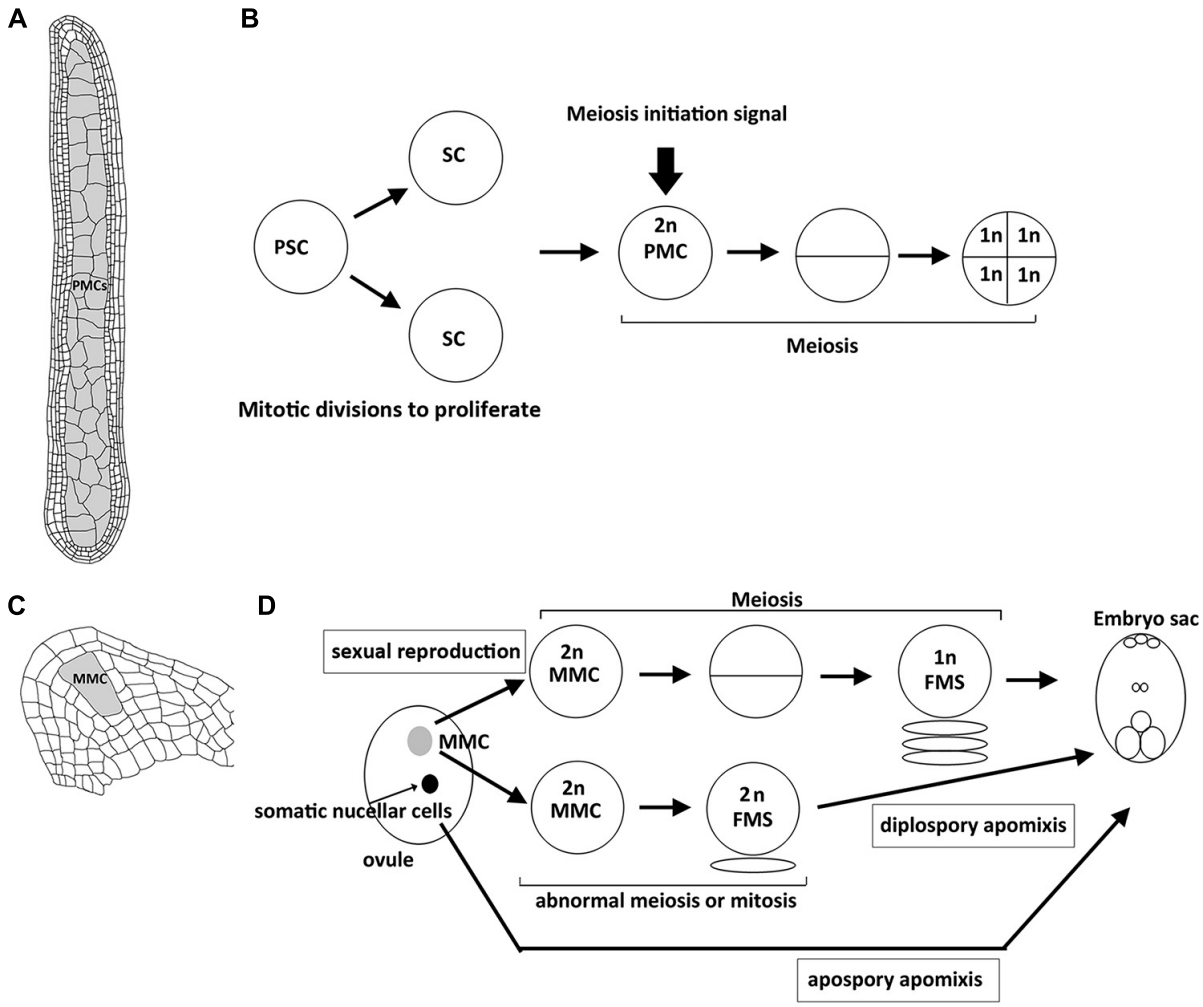
**Structure of plant reproductive organs in maize and sequence of events leading to spore or gametophyte formation in anthers and ovules. (A)** Longitudinal section of an anther with numerous pollen mother cells (PMCs, shown in gray) that are proliferated from primary sporogenous cells by mitosis, which accompanies the development of surrounding 4 layers of somatic cells. **(B)** After primary sporogenous cells (PSCs) are differentiated, they first undergo mitotic divisions to produce sporogenous cells (SCs) and further develop into PMCs. By the time when the development of surrounding somatic cells (shown in **A**) is complete, unknown meiosis initiation siganl is generated to start meisois synchronously in all PMCs of an anther. Each PMC enters meiosis to produce four haploid spore cells. **(C)** Longitudinal section of an ovule with a single megaspore mother cell (MMC, shown in gray). **(D)** Schematic illustration showing the sequential development of embryo sac through sexual reproduction or apomictic pathways. In sexual reproduction, the single MMC (shown in gray) is differentiated and then enters meiosis to produce a haploid functional megaspore (FMS), and then develops into an embryo sac. In diplospory apomixis, the specified MMC unergoes an abnormal meiosis or mitosis to produce a diploid FMS. In apospory apomixis, somatic nucellar cells develop into embryo sac without meiosis.

In plants, meiosis is initiated in sporogenous cells that are differentiated in ovules and anthers (Bhatt et al., 2001). In each ovule, only a single megaspore mother cell (MMC) surrounded by the somatic nucellar cells is differentiated and then undergoes meiosis (**Figure 1**). During anther development, after primary sporogenous cells (i.e., the precursor of pollen mother cells, PMCs) are differentiated, they first undergo several rounds of mitosis to proliferate, and then meiosis occurs synchronously in all the PMCs of each anther (**Figure 1**; Palmer, 1971). Thus, the decision of mitosis–meiosis transition must coordinate with the developmental stages of anthers and ovules. For example, the signal that starts meiosis in an anther must be generated after complete development of the somatic layers of anthers (Kelliher and Walbot, 2011). Interestingly, the signal can also establish the synchronization of the meiotic cell cycle in an anther. On the other hand, only a single MMC in each ovule is specified to enter meiosis, which accompanies the development of ovule in parallel. Thus, the regulatory mechanism of meiosis initiation may be different between female and male in plants because of distinct development of sporogenesis.

The first discovery about meiosis initiation was the isolation of the maize *ameiotic1* (*am1*) mutant by Rhoades (1956. The original *am1* mutant allele does not undergo meiosis; instead mitosis-like divisions take place in well-developed meiocytes in both female and male organs (Golubovskaya et al., 1993). *Am1* encodes a plant specific coiled-coil protein with unknown functions (Pawlowski et al., 2009). All five null mutant alleles display identical phenotypes in male meiocytes in which mitosis replaces meiosis. However, female MMCs in the mutant may either undergo mitosis, or arrest at interphase. Interestingly, the *am1-praI* allele carrying a single amino acid substitution (R358W) can enter meiosis but cells arrest in the leptotene/zygotene stage, resembling the phenotype of the rice *am1* mutant that also carries an amino acid substitution (R360W) in a conserved region (Golubovskaya et al., 1997; Pawlowski et al., 2009; Che et al., 2011). These results suggest that AM1 is required for meiosis initiation and may also regulate meiotic progression. In contrast to maize and rice, mutants in the closest homolog of *Am1* in *Arabidopsis, switch1/dyad* (*swi1*), exhibit abnormal meiosis with sister chromatid cohesion defects in male meiocytes, and the mitosis-like division was only observed in female meiocytes (Mercier et al., 2001, 2003). These differences among species may indicate that the AM1-related genes have undergone species-specific diversification.

While the molecular functions of AM1/SWI1 are still unknown, microarray analyses showed that AM1 is required for normal expression of many meiotic genes (Nan et al., 2011). Using Agilent 44K microarrays, the authors compared transcriptomes in 1-mm and 1.5-mm anthers of *am1-489* (null allele) and *am1-praI* (point mutant allele) and their fertile siblings. In 1-mm anthers when meiosis is about to start in the wild-type, 484 genes were missing and 1208 genes were ectopically expressed in *am1-489* anthers. These genes are considered to contribute to the initiation of meiosis or the suppression of mitosis. In 1.5-mm anthers, during prophase I in the wild-type, 3700 transcripts were missing and another 3107 genes were differentially expressed in *am1-489* anthers. Nearly 60% of transcriptome changes, regardless of stage, were genes enriched in PMCs and many putative meiosis-related genes were found among them. However, none of the meiosis-related genes were regulated in an absolute On/Off pattern on the *am1-489* allele, somewhat surprising given that the *am1-489* PMCs perform mitosis instead of abnormal meiosis. These results redefine the role of AM1 in the modulation of transcript accumulation for many meiotic genes rather than simply switching them on or off (Nan et al., 2011).

Recently, microarray analyses on laser-captured germinal and somatic initials from maize 0.3-mm anthers (right after sporogenous cells are differentiated) found about 2500 genes specific or enriched in germinal initials (Kelliher and Walbot, 2014). Surprisingly, more than 100 meiotic genes are expressed in the mitotic amplification period that is long before the onset of meiosis initiation. This finding raises a possibility that precocious expression of meiotic genes permits gradual dilution of mitotic chromatin components, a hypothesis recently proposed for the mouse germ-line (Hackett et al., 2013). Another possibility is that those PMC precursors are preparing for meiosis at the transcriptional level, and may store some meiotic transcripts for translation at later developmental stages (Zhang et al., 2014). Regardless, this finding suggests that the decision to start meiosis is a series of consecutive steps rather than a single switch. Perhaps, the expression of meiotic genes may be one of the earliest actions, and the following regulatory cascade finally governs the initiation and progression of meiosis. Thus, which transcription factors are responsible for early meiotic gene expression and whether meiotic genes are under translational control are interesting questions for further study. In addition, identification of components in the regulatory cascade will provide better understanding of this process.

Another gene that has been reported to be involved in meiosis initiation is rice *MEL2*, named for its “meiosis arrested at leptotene” phenotype. *MEL2* encodes an RNA-recognition-motif (RRM) protein, and it is required for regulating the premeiotic G1/S-phase transition of male and female germ cells (Nonomura et al., 2011) as most germ cells fail to enter pre-meiotic S phase in *mel2* mutant. A small proportion of PMCs can escape from the defects and undergo meiosis with a significant delay or continued mitotic cycles. How an RRM protein affects the initiation of meiosis is unclear at the molecular level, but this result implied a possible link between mRNA processing, transport or stability, and entry into meiosis in plants. Studies in yeast have shown that the final trigger to start meiosis is the activation of specific cyclin–CDK complexes to initiate the meiotic S phase. *Arabidopsis* has at least 50 cyclins and only a few of them are specifically expressed in the inflorescence (Bulankova et al., 2013). Mutant analyses revealed that some of these cyclins contribute to distinct meiosis-related processes, but none of cyclin mutants showed meiosis initiation defects, which was attributed to gene redundancy. Thus, it will be interesting to know which, if any, cyclin–CDK complex is responsible for the transition. Besides cyclin–CDK complexes, some meiosis-specific regulators, such as replication factor MUM2 and cohesion protein REC8, are involved at the meiotic S phase although much of the basic replication apparatus is employed (Strich, 2004). Therefore, what is special about the pre-meiotic S phase and which are the specific genes that differ from the mitotic S phase in plants? Understanding of these meiosis-specific components at meiotic S phase will help us to illustrate the molecular mechanisms of meiosis initiation. A proteomics study may offer valuable information on this aspect.

To date, mutants directly affecting meiosis initiation showed similar phenotypes in that some of reproductive cells fail to enter meiosis in either female, male, or both sexes. Although some of these mutants produce unreduced daughter cells by mitosis-like division, there is no evidence that these resulting diploid cells in ovules would undergo the apomictic pathway without fertilization. However, an interesting study demonstrated that in the *Arabidopsis swi1/dyad* mutant*,* few seeds were produced when pollinated with wild-type pollens. Most of the progeny were triploid, suggesting that unreduced female daughter cells after mitosis-like division are able to develop further and be fertilized by haploid male gametes (Ravi et al., 2008).

## CURRENT ADVANCES IN THE APOMICTIC PATHWAY

Apomixis is a type of asexual reproduction through seeds that avoid both meiosis and fertilization. In the apomictic pathway, differentiated MMCs or other somatic cells in ovules that gain germinal cell fate are able to bypass meiosis or undergo an abnormal meiosis to produce unreduced spores that further divide mitotically to form an embryo sac (**Figure 1**; Koltunow, 1993; Carman, 1997). Although apomixis is genetically regulated and occurs naturally in more than 400 species of flowering plants, its implementation at the molecular level is still unclear. Over the past few years, there has been increasing evidence to show that epigenetic control may regulate apomixis. In *Arabidopsis, argonaute 9 (ago9)* mutants exhibit multiple MMCs compared to a single MMC in the wild-type ovule, and additional MMCs in the mutant are able to initiate gametogenesis without undergoing meiosis, resembling apospory (**Figure 1**; Olmedo-Monfil et al., 2010). AGO9 preferentially interacts with 24-nucleotide small interfering RNAs (siRNA) derived from transposable elements to direct homolog-based RNA-dependent DNA methylation (RdDM). Moreover, mutations in *SUPPRESSOR OF GENE SILENCING3* (*SGS3*) and *RNA-DEPENDENT RNA POLYMERASE6* (*RDR6*), two genes required for siRNA biogenesis, also lead to an identical defect to that in *ago9* mutants (Olmedo-Monfil et al., 2010). Similarly, maize AGO104, the homolog of *Arabidopsis* AGO9, is found to regulate reproductive fate despite some differences between maize *ago104* and *Arabidopsis ago9* phenotypes (Singh et al., 2011). The maize *ago104* mutant has a single MMC; however, defective female meiosis with aberrant condensation results in functional female gametes with an unreduced chromosome set, resembling diplospory (**Figure 1**). Additionally, AGO104 is required for heterochromatic CHG and CHH methylation. Consistent with the idea that epigenetics regulates apomixis, mutations of two DNA methyltransferases, DMT102 and DMT103 in maize, also exhibit apomictic development (Garcia-Aguilar et al., 2010). Thus, loss of RdDM seems to direct somatic cells to distinct reproductive cells with an apomictic fate (seen in *Arabidopsis ago9* mutant) or lead to apomixis in correctly specified MMCs (seen in maize *ago104* mutant). Interestingly, both AGO9 in *Arabidopsis* and AGO104 in maize are specifically expressed in surrounding somatic nucellar cells, and not in the reproductive cells, implying that both genes control the apomictic pathway in a non-cell-autonomous manner. siRNAs produced from somatic cells may move to germinal cells to regulate the chromatin state by suppressing transposable elements. Indeed, many transposable elements are silenced in *Arabidopsis* wild-type ovules, in an AGO9-dependent manner (Durán-Figueroa and Vielle-Calzada, 2010). These results suggest a link between siRNA-dependent chromatin remodeling and the apomictic pathway (Garcia-Aguilar et al., 2010; Grimanelli, 2012).

Another gene belonging to the ARGONAUTE family with meiotic phenotypes is rice *MEL1*. It encodes an AGO5 protein that is required for maintaining germ cell identity and normal meiosis progression. Interestingly, the *mel1* mutant also shows defective chromosome condensation with abnormal pericentromere histone modification (Nonomura et al., 2007). Recently, MEL1 has been shown to bind to 21-nucleotide phased small interfering RNA (Komiya et al., 2014). Further investigation is needed to understand the epigenetic regulation of plant reproduction.

Over the past few years, the identification of mutants has shed light on genetic control of epigenetic mechanisms involved in apomixis. However, it is still not clear how the RdDM-dependent process affects cell fate specification, meiosis, and gametophyte development? Why is there a need for transposes-derived siRNA in the germ line? Is it possible that RdDM resets cell fate in the germ line, a role also demonstrated for the animal PIWI pathway (Houwing et al., 2008; Juliano et al., 2011)? Perhaps, identifying the targets of the RdDM pathway at different stages will be essential for further definition of their roles. In addition, what is the relationship between AM1/SWI1-dependent meiosis initiation and the RdDM pathway? It is worth noting that alterations in histone modification were observed in the *swi1* mutant (Boateng et al., 2008), raising the possibility that somehow AM1/SWI1 is involved in chromatin remodeling. Many exciting questions are awaiting further investigation.

## CONCLUSION

Understanding the initiation of meiosis and apomixis in plants will be enlightening, and may have many potential applications for plant breeding and in agriculture including developing a strategy for acquiring apomixis in crops, and allowing manipulation of the meiotic cell cycle. It will be crucial to identify more participants in the mitosis–meiosis decision and the apomictic pathway and to explore their molecular functions.

## Conflict of Interest Statement

The authors declare that the research was conducted in the absence of any commercial or financial relationships that could be construed as a potential conflict of interest.
